# *Astragalus* polysaccharide enhances immunity and inhibits H9N2 avian influenza virus *in vitro* and *in vivo*

**DOI:** 10.1186/2049-1891-4-22

**Published:** 2013-06-21

**Authors:** Sanpha Kallon, Xiaorong Li, Jun Ji, Cuiying Chen, Qianyun Xi, Shuang Chang, Chunyi Xue, Jingyun Ma, Qingmei Xie, Youngliang Zhang

**Affiliations:** 1 College of Animal Science, South China Agricultural University, Guangzhou 510642, China; 2 US Department of Agriculture, Agricultural Research Service, Avian Disease and Oncology Laboratory, 3606 East Mount Hope Road, East Lansing, MI 48823, USA; 3 State Key Laboratory of Biocontrol, College of Life Sciences, Sun Yat-Sen University, Guangzhou 510006, China

**Keywords:** *Astragalus* polysaccharide, H9N2 avian influenza virus, Immune effect

## Abstract

This study investigated the humoral immunization of *Astragalus* polysaccharide (APS) against H9N2 avian influenza virus (H9N2 AIV) infection in chickens.

The effects of APS treatment on H9N2 infection was evaluated by an MTT [3(4, 5-dimethylthiazol-2-yl)-2, 3-diphenyl tetrazolium bromide] assay and analysis of MHC and cytokine mRNA expression. The effect on lymphocyte and serum antibody titers *in vivo* was also investigated. IL-4, IL-6, IL-10, LITAF, IL-12 and antibody titers to H9N2 AIV were enhanced in the first week after APS treatment. The results indicated that APS treatment reduces H9N2 AIV replication and promotes early humoral immune responses in young chickens.

## Background

Avian influenza (AI) is a highly contagious disease which causes acute illness, often culminating in death, in domestic poultry and other animals. The high morbidity and mortality associated with this pathogen have an impact on both economic and societal costs. Migratory birds, mechanical vectors (such as contaminated cages and clothing), and the international trade in live poultry, contribute to the spread of infection. The disease can be transmitted to humans through exposure to infected birds or the handling of infected carcasses [1]. The disease has wide occurrence and the virus has the potential to mutate to a highly pathogenic form with the potential for zoonotic transmission. AI subtype H9N2 belongs to the low pathogenic avian influenza virus (AIV) group; considered to be a common cause of disease epidemics [2,3]. H9N2 infections in chickens are associated with low mortality rates, mild respiratory infections, reduced performances in egg production (particularly for the local poultry industry), and co-infections with *Staphylococcus aureus* and *Haemophilus paragallinarum*, or attenuated vaccine strains which exacerbate the disease [2]. Currently, vaccination is one of the most promising control measures for H9N2 AIV. Unvaccinated chickens can become infected with modified live H9N2 virus strains used for early immunization [4], while inactivated vaccines have been shown to be safe and efficacious against H9N2 AIV [5]. Novel immune adjuvants are still in development and their use is highly desirable for producing vaccines which evoke a rapid immune response against viruses.

Compositional analysis by gas chromatography revealed that Chinese herbal polysaccharides such as *Astragalus* [Huang qi] contain mannose, D-glucose, D-galactose, xylose and L-arabinose. These polysaccharides are used as an immunomodulating agent in mixed herbal decoctions to treat common colds, diarrhea, fatigue, and anorexia [6].

In China, APS is widely used as an immune adjuvant; having been identified as a class of macromolecule that can profoundly affect the immune system, stimulate cell proliferation, induce the expression of surface antigens on lymphocytes, affect the expression of cytokines, and promote the production of antibodies [7]. In a previous study, it was reported that APS possess effective immunostimulatory effects when used in vaccination programs against Foot and mouth disease virus (FMDV), Newcastle disease virus (NDV) and Infectious bursal disease virus (IBDV) [5,8]. The appropriate dose of APS was likely to increase the expression of MHC class II, CD40, and CD86, and improve FMDV antigen-presentation during the early stages of the immune response [8].

In this research, the appropriate concentration and antiviral action of APS on the propagation of H9N2 AIV in chick embryo fibroblasts (CEF) was investigated. We also studied how APS affected mRNA expression of IL-2, IL-4, IL-6, IL-10, LITAF and IL-12 in CEF. The variation in peripheral blood lymphocytes in chickens before and after immunization, and in antibody titer, were also investigated to assess the immunoregulatory effect of APS on chickens at pre-vaccination, and to evaluate the immunization potential of *Astragalus* polysaccharide (APS) against H9N2 AVI.

## Materials and methods

### Preparation of H9N2 avian influenza virus and cell culture

Ten-day-old embryonating specific-pathogen-free (SPF) chicken eggs (Guangdong Dahuanong Animal Health Products Co. Ltd, Guangzhou, China) were inoculated with H9N2 virus (0.2mL/egg). Infected allantoic fluids were harvested 48h post-inoculation and concentrated with a 100K tangential flow filtration capsule (Pall Life Sciences) by centrifugation at 40,000rpm for 1h. The suspension was loaded onto a 30 to 60% (wt/wt) sucrose gradient and subjected to centrifugation at 26,000rpm at 4°C with a SW-40 Ti rotor (Beckman Instruments, Palo Alto, CA) for 3h using the slowest acceleration and braking rates. The viral pellets were washed and centrifuged at 40,000rpm for 1h at 4°C. Subsequently, the pellets were re-dissolved in 1mL of PBS, filtered through a 0.22 Millipore filter, and stored at −70°C [9].

CEF cultures were prepared from 10-day-old chicken embryos according to standard protocols [10,11]. Dulbecco’s Modified Eagle Medium (DMEM; Gibco-Invitrogen) was used as the growth medium. In brief, embryo tissue was cut into pieces and diluted to 1 × 10^6^ cells/mL Following filtration the cells were cultivated in a 5% CO_2_ incubator at 37°C for 48h.

### Extraction and purification of APS

Powder ground APS obtained from South China Agricultural University (Guangzhou, China) was boiled in distilled water for 4h at 100°C. After filtration to remove debris, the filtrate was concentrated in a rotary evaporator. Protein was removed using the Savage method [12]. Crude polysaccharide fractions were obtained by precipitation with three volumes of ethanol and desiccation *in vacuo*. The precipitate was re-dissolved in distilled water and loaded on a D101 macro-porous resin column (2.6cm × 60cm) to remove pigment. The effluent was collected and the polysaccharide fractions were quantitatively determined using a phenol-sulfuric acid assay [13] with glucose as the reference standard.

### Determination of safety concentration of APS to CEF

The CEF safety concentration was determined as reported by Huang et al. [14]. In brief, CEF monolayers in 96-well plates were exposed to *Astragalus* polysaccharide at a series of concentrations; conducted in replicates of four wells per concentration. After culturing for 72h at 38.5°C in a humidified atmosphere of 5% CO_2_, the supernatant was removed and 100μL Dimethyl sulfoxide (DMSO; Sigma, Kent, UK) added. The plates were shaken for 5min to ensure complete dissolution of the crystals. The absorbance at 570nm (A_570_) for each well was measured by a microtiter enzyme-linked immunosorbent assay reader (Model DG-3022, East China Vacuum Tube Manufacturer). The A_570_ value correlates to the number of live cells; the higher the value at A_570_ the greater the number of viable cells. The A_570_ values for APS treated CEFs were higher than for the corresponding cells of the control group. These results indicated that the polysaccharide was not cytotoxic to CEFs at the concentrations used. The corresponding concentrations of APS were considered as the maximum safety concentration for CEF.

### Determination of optimal APS concentration for CEF growth

CEF confluent monolayers, prepared in sextuplicate in 96-well culture plates, were overlaid with serial doubling dilutions of APS in DMEM (2,500 to 4.833μg/mL), and cultured at 37°C in a humidified 5% CO_2_ incubator for 8h. The effect of APS concentration on CEF viability was determined by the MTT 3-(4,5-Dimethyithiazol-2-yl)-2, 5-diphenyltetrazolium bromide) assay. Briefly, the media was discarded, 20μL of MTT (Amresco Solon, OH; 5 ìg/mL) added to each well, and the plates incubated at 37°C in a humidified atmosphere of 5% CO_2_ for 4h. At the completion of the incubation, 10μL DMSO (Sigma) was added and the plates incubated at 37°C for 5min to ensure complete dissolution of the formazan crystals. Absorbance was measured at A_570_ using a microtiter ELISA reader (Molecular Devices Emax, CA, USA).

### Antiviral assays

Antiviral activity was evaluated as reported by Huang et al. [14]. In brief, serial two-fold dilutions of APS and H9N2 virus were added to monolayers of CEF cultures under the conditions detailed below.

Pre-addition of APS: APS solutions (100mL/well, four wells per concentration) were added to CEF monolayers prior to the addition of virus. Following incubation in APS for 2h at 38.5°C with 5% CO_2_, the supernatant was removed, the cells washed twice with Hanks’ solution, and the virus added.

Post-addition of APS: The viral solutions were added to CEF monolayers prior to the addition of APS. Following incubation with H9N2 for 2h at 38.5°C with 5% CO_2_, the viral inoculum was removed, the cells washed twice with Hanks’ solution, and APS solution added; four wells for each concentration.

Simultaneous addition of APS and H9N2 virus: APS and virus solutions were pre-incubated together for 4h at 4°C prior to addition to CEF monolayers; four wells per concentration.

All plates were cultured at 38.5°C in a 5% CO_2_ incubator. When complete cytopathic effect (CPE) was observed for the H9N2 control group (~ 72h), CEF viability was measured by MTT assay. The mean cellular A_570_ values were an indicator of antiviral activity. When the A_570_ value of the APS group was significantly higher than that of the virus control group, it demonstrated that the corresponding polysaccharide had significant activity.

### Animal experiments

One hundred and twenty, five-day-old SPF white male leghorn avian broilers, purchased from Guangdong Dahuanong Animal Health products Co. Ltd., were divided into four groups and maintained in four positive pressure isolators. Experimental groups contained three sample groups (30 chickens per group) and one control group (including vaccinated and unvaccinated).

At 7 d post hatch, three sample groups were hypodermically injected with APS at three different concentrations (5mg/kg, 10mg/kg and 20mg/kg) once a day for five successive days. At 12 d post-hatch, six chickens per group were euthanized by cervical dislocation and their blood immediately collected. Peripheral blood lymphocytes separated from the blood samples (5mL per chicken) were used for flow cytometry and real-time PCR. The immune organs were weighted for statistical analysis and immediately stored at −70°C. Simultaneously, nine chickens per group were challenged with 10^7.5^EID_50_/0.2mL of H9N2 AIV. Additionally, 15 birds were taken from each group, with the exception of the control group, and were immunized subcutaneously with an inactivated oil-emulsion vaccine of H9N2 avian influenza virus (Guangdong Dahuanong Animal Health Products Co. Ltd.) at 13 and 20 d post-hatch. Antibody titers were measured at seven and 14 d post-immunization. The experimental procedures performed on chickens at 12 d post-hatch were repeated at 26 d post- hatch.

### Ethics statement

All animal experiments and husbandry involved in this study, and presented in this manuscript, were conducted in accordance with the guidelines of the South China Agricultural University Animal Care and Use Committee, which operates under the Animal Welfare Law and Regulations of the Department of Health and Human Services. The South China Agricultural University Animal Care and Use Committee approved all details of this study.

### Three color flow cytometry

Blood samples were placed in Petri tubes with sodium heparin and intermixed with an equal volume of Hanks’ balanced salt solution (HBSS, pH 7.2 ~ 7.4). An aliquot was layered over 5mL LSM (lymphocyte separation medium) and centrifuged at 2,000rpm for 20min in a swing-bucket rotor. Peripheral blood lymphocytes were recovered from the LSM-HBSS interface and washed 3 times with PBS. A total of 2 × 10^6^ lymphocytes inrpmI 1640 medium (Roswell Park Memorial Institute 1640 medium) containing 10% (vol/vol) BSA were incubated with 20μL anti-chicken CDS-FITC (SouthernBiotech) in the absence of light for 20min. Cell suspensions were washed with 500μL PBS, centrifuged at 1,200rpm for 10min, the supernatant discarded and the cells re-suspended in 500μL PBS [15]. The cells were then analyzed using a FACSAria flow cytometer (version 6.1; BD Biosciences).

### Measurement of antibody titer

The antibody titer was measured by the hemagglutination inhibition (HI) test [16]. The HI test was a standard beta test, using 4 hemagglutinating units of antigen in 96-well plates, where the test serum had been diluted two-fold. HI endpoint titers were determined as the reciprocal of the highest serum dilution that produced complete inhibition of hemagglutination.

Blood samples (1.0mL/chicken) obtained from the main brachial vein were drawn into Eppendorf tubes and allowed to clot at 37°C for 2 h. Serum was separated by centrifugation and stored at −20°C for HI antibody determination. Briefly, two-fold serial dilutions (1:2 to 1:2,048) of heat inactivated (56°C for 30min) serum, was applied to a 96-well, V-shaped bottom microtiter plate containing 50μL/wellcmF-PBS, and 50μL/well H9N2 antigen (4 hemagglutination units); with the exception of the last row (controls). The antigen: serum mixture was incubated for 10min at 37°C, 50μL of a 1% rooster erythrocyte suspension added to each well and the plates re-incubated for 30min. Positive serum, negative serum, erythrocytes, and antigens were also included as controls. The highest dilution of serum causing complete inhibition of erythrocyte agglutination was considered the end point. The geometric mean titer was expressed as reciprocal log_2_ values of the highest dilution that displayed anti-H9N2-HI.

### Real-time PCR

Total RNA was extracted from CEF using Trizol reagent (Takara Biotechnology, Dalian, China) for the detection of cytokines and H9N2 expression. The quantity of total RNA was measured by ultraviolet spectrophotometry and the optical density (OD) _260/280_ determined to be between 1.8 and 2.0. The isolated RNA was digested with Recombinant DNase I (Takara Biotechnology, Dalian, China) at 37°C for 30min. Oneμg of total RNA was used for reverse transcription with ReverTra Ace® qPCR RT kit (Toyobo; Osaka, Japan) and amplifications were performed with 1μL cDNA in a total volume of 20μL SYBR Green Real-time PCR Master Mix (Roche; Mortlake, Australia); all reactions were performed with the Stratagene Mx3005P QPCR system (La Jolla, Ca) according to the manufacturer’s instruction. All reactions were completed in triplicate. Relative expression fold change was calculated by the 2^△△Ct^ method, and glyceraldehyde-3-phosphate-dehydrogenase (GAPDH) was used as the endogenous reference gene to normalize the expression level of target gene. The primers used in the real-time PCR assay were listed in Table 1.

**Table 1:** Sequences of the oligonucleotide primers used in real-time quantitative PCR

**RNA target**	**Primer sequences**		**Size of PCR product, bp**
	**Sense**	**Anti-sense**	
MHC class I*	5′-AAGAAGGGGAAGGGCTACAA-3′	5′-AAGCAGTGCAGGCAAAGAAT-3′	222
MHC class II*	5′-CTCGAGGTCATGATCAGCAA-3′	5′-TGTAAACGTCTCCCCTTTGG-3′	312
Interferon γ	5′-TGAGCCAGATTGTTTCGA-3′	5′-ACGCCATCAGGAAGGTTG-3′	118
LITAF	5′-TTCTATGACCGCCCAGTT-3′	5′-CAGAGCATCAACGCAAAA-3′	165
IL-2*	5′-CGGGATCCATGATGTGCAAAGTACTG-3′	5′-CGGTCGACTTATTTTTGCAGATATCT-3′	80
IL-4	5′-GAGAGGTTTCCTGCGTCAAG-3′	5′-TGACGCATGTTGAGGAAGAG-3′	76
IL-6	5′-ATAAATCCCGATGAAGTGG-3′	5′-CTCACGGTCTTCTCCATAAA-3′	146
Il-10	5′-CAATCCAGGGACGATGAAC-3′	5′-GCAGGTGAAGAAGCGGTGA-3′	94
Il-12	5′-GGGAACAGAACTGAAAGG-3′	5′-GCTGATAATCTCGTGGG-3′	109
H9N2	5′-ATGCGGTGGAAGATGGG-3′	5′-AGGCGACAGTCGAATAAATG-3′	198
GAPDH*	5′-CCTCTCTGGCAAAGTCCAAG-3′	5′-CATCTGCCCATTTGATGTTG-3′	200

MHC = Major histocompatibility complex.

GAPDH = glyceraldehyde-3-phosphate-dehydrogenase.

### Statistical analysis

All data were presented as mean ± SEM. Comparisons between two groups were analyzed using unpaired Student’s T- tests. Comparisons among multiple groups were analyzed by ANOVA followed by post hoc analysis using the Tukey’s multiple comparison test. All statistical analysis was conducted using SPSS 17.0 software (SPSS, Chicago, IL, USA) and *P* < 0.05 was considered to be statistically significant. All experiments were performed at least three times.

## Results

### Optimal APS concentration and antiviral assays

Determination of the optimal APS concentration and the results of the antiviral assays are presented in Tables 2 and 3. The optimal APS concentration for the stimulation of CEF proliferation was determined to be 321.25μg/mL. An increase in CEF proliferation was observed for all APS concentrations studied (4.833 to 2,500μg/mL).

**Table 2:** **The A**_**570 **_**values of the optimal APS concentration and antiviral assays (25006μg/mL −156.6μg/mL)**

**Groups**	**Groups**				
	**2,500μg/mL**	**1,250μg/mL**	**625μg/mL**	**321.25μg/mL**	**156.6μg/mL**
APS	0.281 ± 0.026^abcd*^	0.303 ± 0.0061^abc*^	0.304 ± 0.019^abc*^	0.314 ± 0.017^ab*^	0.308 ± 0.017^abc*^
Pre-add APS	0.237 ± 0.003^a*^	0.238 ± 0.004^a*^	0.241 ± 0.012^a*^	0.269 ± 0.016^b*^	0.266 ± 0.006^ab*^
Post-add APS	0.205 ± 0.011^abc^	0.204 ± 0.004^bd^	0.198 ± 0.006^de^	0.190 ± 0.004^de^	0.178 ± 0.013^de^
Sim-add APS	0.166 ± 0.004^a^	0.174 ± 0.005^a^	0.181 ± 0.002^ab^	0.182 ± 0.004^abcd^	0.183 ± 0.007^be^
Virus Control	0.175 ± 0.007				
Cell Control	0.242 ± 0.002*				

^a-f^ data within row without the same superscripts differ significantly (*P* < 0.05). *Data differed significantly (*P* < 0.05) when compared with virus control.

APS = *Astragalus* polysaccharide.

**Table 3:** **The A**_**570 **_**values of the optimal APS concentration and antiviral assays (78.216 and 4.83μg/mL)**

**Groups**	**APS concentration**				
	**78.21μg/mL**	**39.09μg/mL**	**19.31μg/mL**	**9.266μg/mL**	**4.83μg/mL**
APS	0.289 ± 0.0081^abcd*^	0.281 ± 0.007^abcde*^	0.265 ± 0.005^df*^	0.264 ± 0.002^cf*^	0.243 ± 0.007^ef*^
Pre-add APS	0.254 ± 0.012^ab*^	0.249 ± 0.005^ab*^	0.252 ± 0.006^ab*^	0.246 ± 0.001^ab*^	0.249 ± 0.002^ab*^
Post-add APS	0.189 ± 0.008^e^	0.181 ± 0.006^de^	0.176 ± 0.007^abd^	0.177 ± 0.006^abc^	0.155 ± 0.008^ac^
Sim-add APS	0.189 ± 0.006^abcd^	0.180 ± 0.004^abcd^	0.171 ± 0.003^def^	0.162 ± 0.004^df^	0.161 ± 0.005^f^
Virus Control	0.175 ± 0.007				
Cell Control	0.242 ± 0.002^*^				

^a-f^ data within row without the same superscripts differ significantly (*P* <0.05). *Data differed significantly (*P* < 0.05) when compared with virus control.

APS = *Astragalus* polysaccharide.

**Figure 1:**
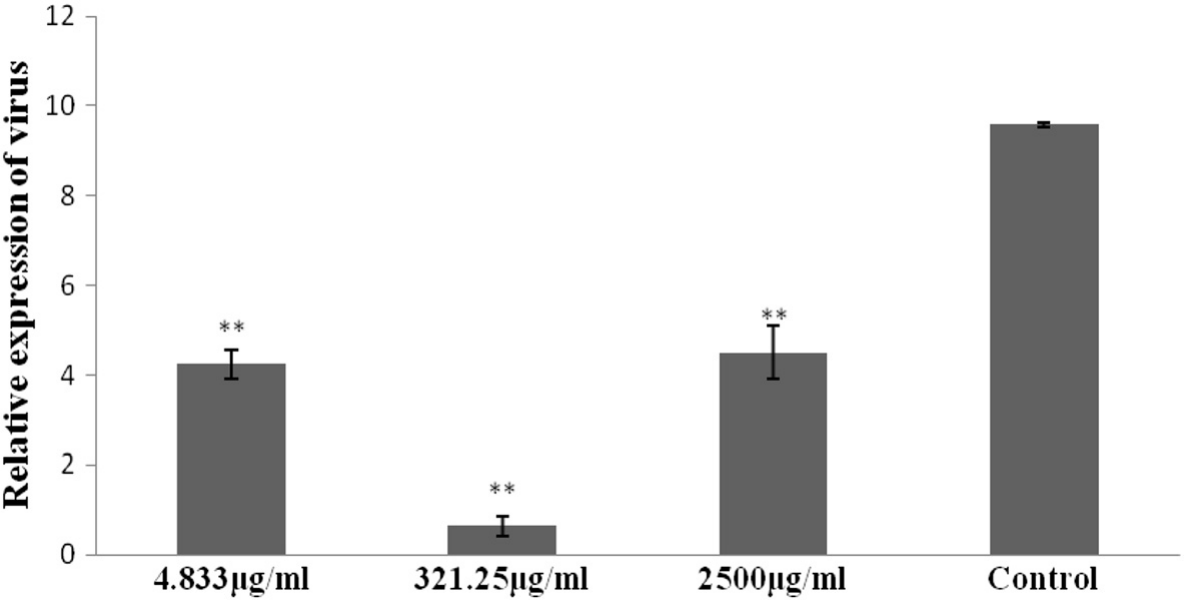
**Illustrates the relative expression of H9N2 virus in CEF at various APS concentrations for the pre-addition APS group.** The inhibition efficiency of APS was more significant at 321.23μg/mL than for 2,500 and 4.833μg/mL.

On pre-treatment of CEF with APS, the A_570_ values for the seven concentrations (4.833 to 321.25μg/mL) were significantly (*P* < 0.05) greater than was observed for the corresponding controls. These results suggest that at these concentrations of APS, CEF proliferation was enhanced, with the greatest degree of proliferation achieved at an APS concentration of 321.25μg/mL. The A_570_ values for both APS and the pre-addition of APS were significantly (*P* < 0.05) higher than for the corresponding virus controls, which indicated that virus activity was inhibited at all APS and pre-addition APS concentrations. In contrast, a number of the A_570_ values for post-addition and simultaneous addition of APS were less than for the virus controls suggesting that at such concentrations viral activity was not inhibited (Tables 2 and 3). For the pre-addition of APS the relative expressions of H9N2 virus at various APS concentrations, particularly 321.25μg/mL, were significantly low. The proliferation of H9N2 was approximately four times lower for the pre-addition APS concentration of 321.25μg/mL when compared with 2,500μg/mL and 4.833μg/mL (Figure 1).

### Major histocompatibility complex and cytokine expression in CEF cells

**Figure 2:**
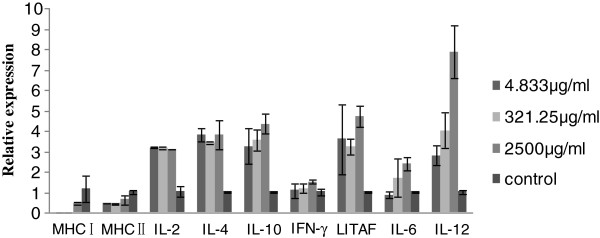
**Illustrates the real-time quantitative PCR analysis of major histocompatibility complexes (MHC) I, MHC II, and cytokines IL-2, IL-4, IL-10, IFN-γ, LITAF, IL-6 and IL-12 in *Astragalus *polysaccharide-treated chicken embryo fibroblasts.** Expression levels of IL-2, IL-4, IL-10, LITAF and IL-12 were higher in the *Astragalus* polysaccharide treatment groups than for the corresponding controls. IL-12 attained the highest expression level.

**Figure 3:**
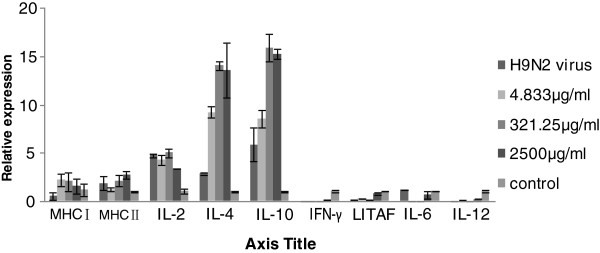
**Illustrates real-time quantitative PCR analysis of major histocompatibility complexes (MHC) I, MHC II, and cytokines IL-2, IL-4, IL-10, IFN-γ, LITAF, IL-6, and IL-12 in *Astragalus *polysaccharide-treated chick embryo fibroblasts (CEF) infected with H9N2 AIV.** IL-4 and IL-10 were expressed to high levels for CEF treated with 321.25μg/mL APS.

The relative expression levels for cytokines IL-4, IL-10, LITAF and IL-12 were high, in particular for IL-12, following APS treatment. Following challenge with H9N2 virus the relative expressions of IL-4 and IL-10 were also considerably high, but the expression of IFN-γ, LITAF, IL-6 and IL-12 were down regulated. MHC I and MHC II expression wasminimal following APS treatment but increased after H9N2 infection (Figures 2 and 3).

### Differential induction of CD3+, CD4+ and CD8+ T Cells

**Figure 4:**
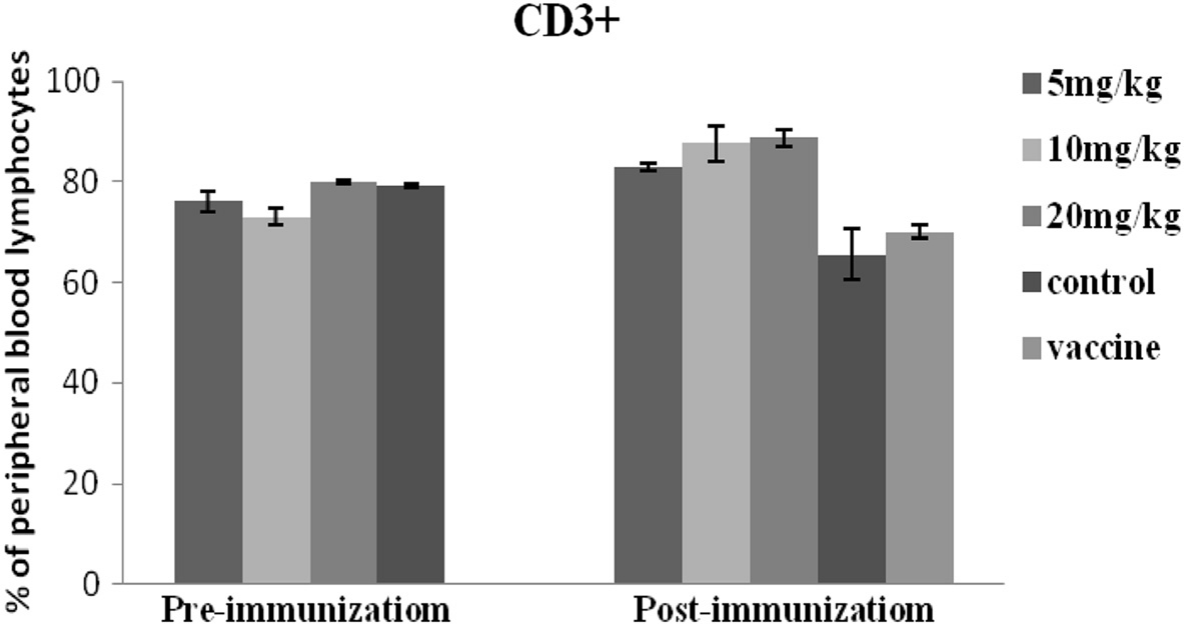
**Illustrates the percentages of CD3+ lymphocyte expression before and after immunization *in vivo*.** Prior to immunization there was no difference in the percentile level of CD3+ for APS treatment groups and control groups. After immunization the CD3+ percentage for the APS groups was higher than for the corresponding controls.

**Figure 5:**
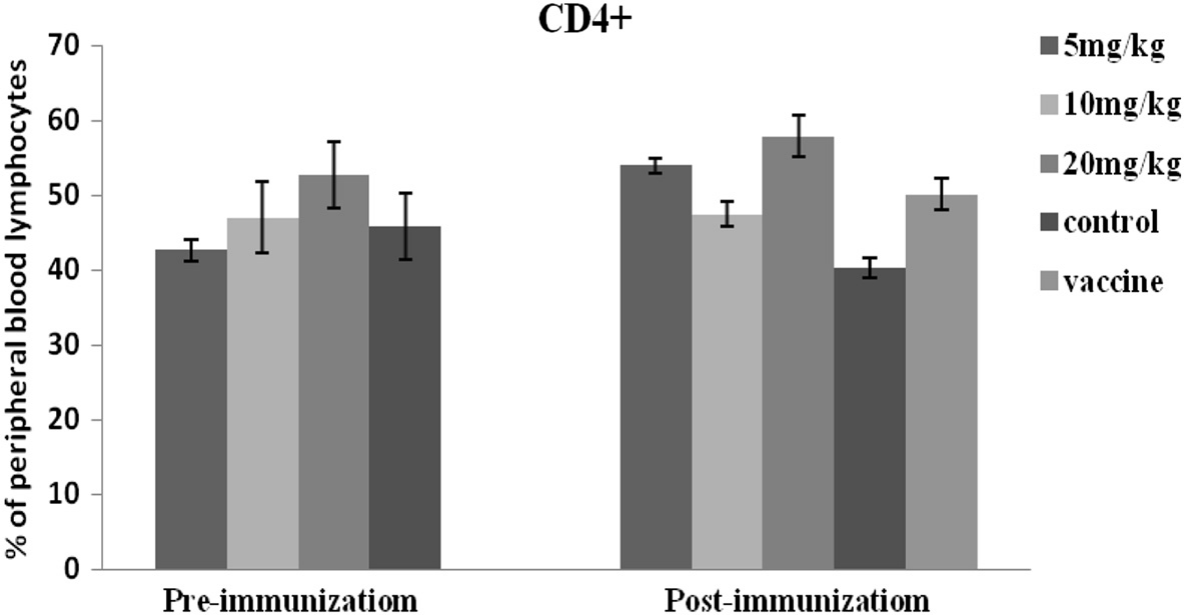
**Illustrates the percentages of CD4 + T cell lymphocyte expression before and after immunization *in vivo. ***CD4+ expression was greatest for the 20mg/kg APS group, both before and after immunization. CD4+ expression increased post immunization for the 5mg/kg APS group.

**Figure 6:**
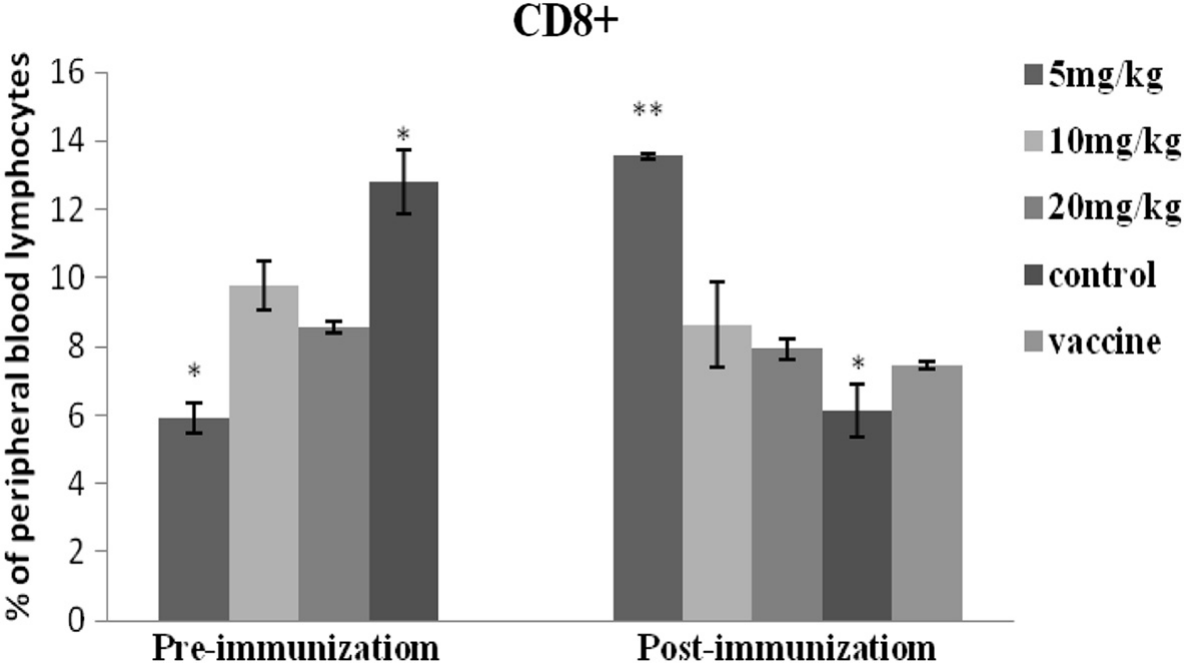
**Illustrates the percentages of CD8 + T cell lymphocyte expression before and after immunization *in vivo*.** CD8+ expression for the 5mg/kg APS group was significantly lower prior to immunization, but increased to levels higher than all the other groups post-immunization.

The proportion of lymphocytes expressing CD3+, CD4+, and CD8+ T cell surface markers are presented in Figures 4, 5 and 6. Prior to immunization, the number of CD3+ and CD8+ lymphocytes were lower for the APS treatment groups (5mg/kg and 10mg/kg), compared with the controls. However, following immunization CD3+ and CD8+ T cell populations increased for the APS treatment groups but decreased for the non-APS treatment groups (Figures 4 and 6). The numbers of CD4+ lymphocytes in the 20mg/kg APS treatment group were higher than for all other groups, both pre- and post- immunization (Figure 5).

### Antibody levels

**Figure 7:**
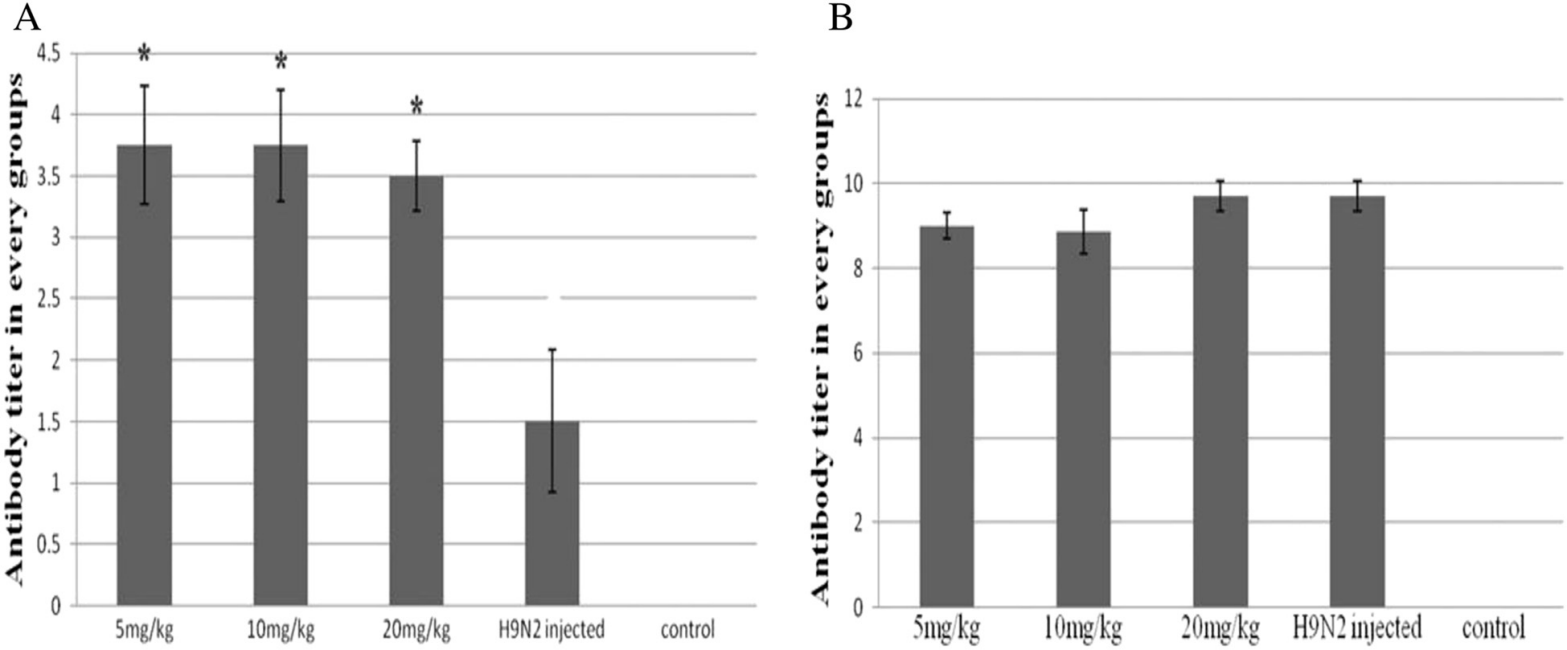
**Antibody titers of chickens at 7 and 14 d post H9N2 AVI infection for different *Astragalus *polysaccharide concentrations.** Antibody titers were greatest for chickens in the 5mg/kg group at seven days post H9N2 infection.

**Figure 8:**
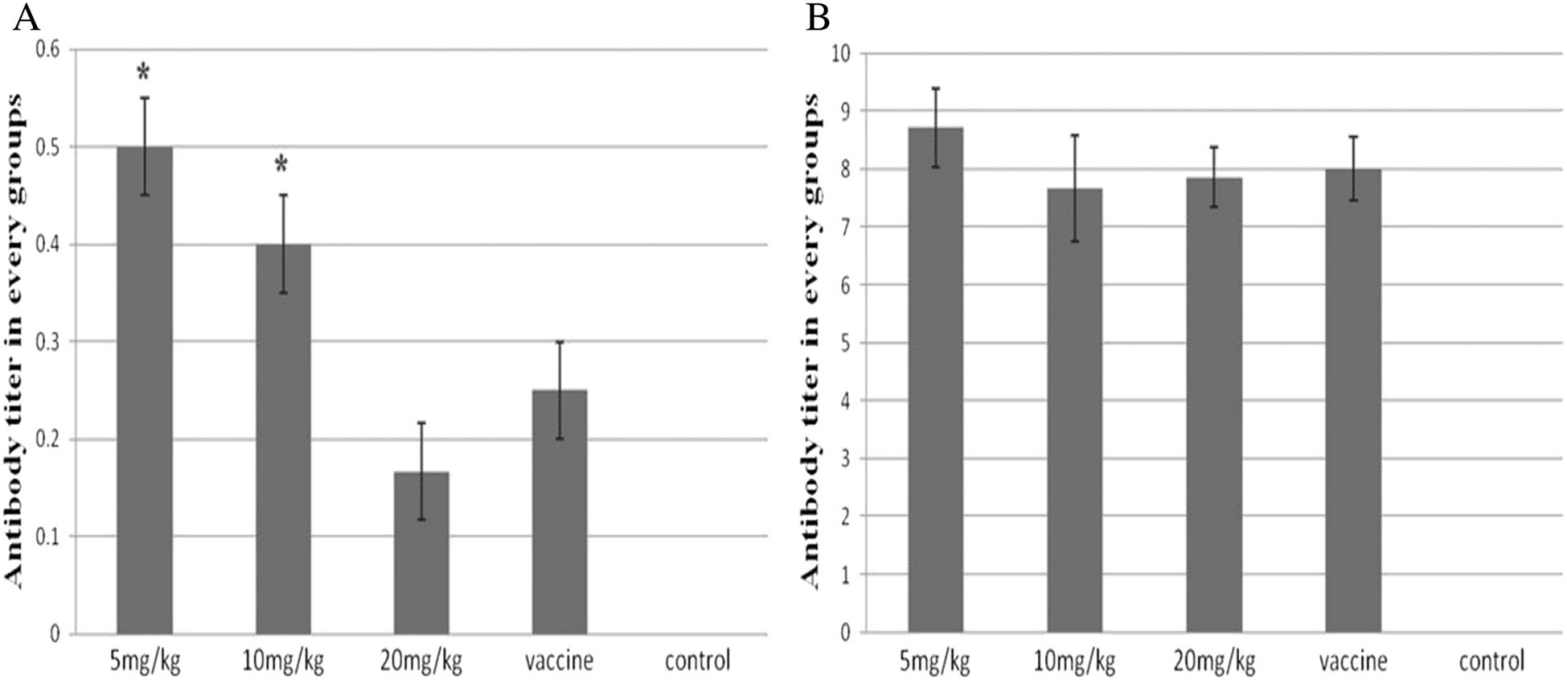
**Antibody titers of chickens at 7 and 14 d post-vaccination with the H9N2 vaccine for different APS concentrations.** Antibody titers were greatest for chickens in the 5mg/kg group at both 7 and 14 d post- vaccination with the H9N2 vaccine.

Antibody levels for the APS treatment groups were significantly (*P* < 0.05) increased, and at a greater rate, than was observed for the untreated group (no APS) seven days post-challenge with H9N2 virus (Figure 7A). Fourteen days post-challenge no difference in antibody titers was observed between the 20mg/kg APS and H9N2 groups (Figure 7B). At seven days post-immunization with inactive vaccine the antibody titers of the APS groups (5mg/kg and 10mg/kg) were significantly (*P* < 0.05) higher than for the untreated groups (Figure 8A). However, at 14 d post-immunization with the inactive vaccine there was no difference in antibody titers between APS treated groups (10mg/kg and 20mg/kg) and the vaccine group (Figure 8B). The 5mg/kg APS group attained the highest antibody titer at 7 and 14 d post-immunization with inactive vaccine (Figures 8A and 8B).

## Discussion

The experimental results revealed the range of optimum APS concentrations for use in studies with chick embryo fibroblasts (CEF). The A_570_ value is an index reflecting the number of living cells, cell growth, and polysaccharide inhibition of virus infection [17]. Higher CEF proliferation was observed in the presence of 321.25μg/mL APS, compared with control groups. Based on these observations and the practicability of APS administration all subsequent *in vitro* experiments were carried out using 4.833, 321.25 and 2,500μg/mL APS. The optimum concentration for APS treatment of CEF was established as 321.25μg/mL. Cell proliferation was enhanced in the presence of 321.25μg/mL APS (compared with the control group), and the cell viability of H9N2 virus infected cells was greater at this concentration of APS than for the other concentrations investigated. When APS was pre-added to cultures of CEF, the A_570_ values for groups within the range 4.833 to 2,500μg/mL APS were significantly higher than for the corresponding virus control group; indicative that APS could prevent H9N2 infection at the concentrations studied. During the simultaneous addition of pre-mixed polysaccharide with virus, the A_570_ values of APS for five concentration groups were significantly larger than for the corresponding virus control groups, which indicated that under these conditions APS could also inhibit H9N2 infection. Following the post-addition of polysaccharide, the A_750_ values for seven APS concentration groups were significantly larger than that of the virus control group; indicative that they too could inhibit H9N2 infection under these conditions (Tables 2 and 3). These results confirmed that when administered at an appropriate dosage, APS had significant antiviral properties, and that the mode of application could enhance the antiviral activity of APS. The real-time PCR results showed that the proliferation of H9N2 virus in CEF was lower for three concentrations in the pre-addition of APS groups, and in particular virus proliferation was significantly low at an APS concentration of 321.25μg/mL (Figure 1). These results demonstrate that APS can protect CEF cells against H9N2 virus infection.

CD3+ and CD4+ are important T lymphocyte markers. CD + T lymphocytes are primarily responsible for mediating cytotoxic effects, and the main function of CD4+ T lymphocytes is the secretion of cell factors which induce and enhance the immune response [18]. In the current study, CD3+ and CD8+ levels increased post- immunization for the APS treated groups, but decreased for the untreated (No APS) groups. Prior to immunization CD4+ lymphocyte populations in the APS treatment groups (5 and 20mg/kg) were greater than for the untreated control (No APS). At both pre- and post-immunization the levels of CD4+ for the 20mg/kg APS treated group was higher than for all other groups; while CD4+ levels for the 5mg/kg APS treatment group increased post-immunization.

In the current study, MHC class I and II were poorly expressed following APS inoculation, but increased upon challenge with H9N2 virus. MHC molecules are responsible for binding to degraded peptides of invading pathogens, their subsequent presentation on the surface of the infected cell for T cell (T_h_ and cytotoxic T cell) recognition, and ultimately for the ensuing destruction of the infected cell. MHC II molecules interact predominantly with CD4+ helper T cells (T_h_), while MHC I molecules interact with CD8+ cytotoxic (killer) T cells. Helper T cells help to trigger an appropriate adaptive immune response. The T cell response is described by the total T cell count (CD3+) and T cell subsets (CD4+ helper and CD8+ cytotoxic cells) [19]. Endogenous antigen is presented by MHC class I molecules and activates CD8+ cytotoxic lymphocytes (CTL); while exogenous antigen is presented by MHC II molecules, activating antibody secreting B cells and helper CD4 + T cells [20]. Following H9N2 infection, increased levels of MHC class I and II were detected in APS treatment groups, when compared with untreated (No APS) groups. Similarly, the percentage of CD3+ and CD8+ was increased; coincident with the up-regulation of IL-4 and IL-10 expression. These observations suggest that APS boosts both T cells and B cells. IL-2 and IFN-γ are secreted by T_h1_ cells and promote cell-mediated immunity [21], whereas IL-2, IL-4, and IL-10 are produced by T_h2_ cells, and activate B lymphocytes resulting in the up regulation of antibody production [21,22]. IL-12 plays an important role in the activities of natural killer (NK) cells, and T-lymphocytes and mediates the enhancement of the cytotoxic activity of NK cells and CD8+ cytotoxic T lymphocytes. It is involved in the differentiation of naïve T cells into Th1 cells. It is known as a T cell-stimulating factor, which can stimulate the growth and function of T cells [23]. It stimulates the production of interferon-gamer (IFN-γ) and tumor necrosis factor-alpha (TNF-α) from T and natural killer (NK) cells, and reduces IL-4 mediated suppression of IFN-γ.

The immune system, as a complex network designed, protects the host from both external (such as bacteria and viruses) and internal threats (such as malignant transformation). Cytokines are important mediators of immune responses. (Role of cytokines in the immune system). The levels of IL-2, IL-4, IL-10 and LITAF expression in APS treatment groups was greater than for the control group; suggesting that APS most probably stimulated cytokine production through B lymphocyte and macrophage activation. Some linear antigens, not readily degraded in the body, and which had an appropriately spaced, highly repeated antigenicity such as the pneumococcal polysaccharide, may stimulate B cells directly; independent of thymus and T cell involvement [24]. Evidence suggests that APS may also be another example of a linear antigen; persisting for long periods on the surface of specialized macrophages situated at the subcapsular sinus of the lymph nodes and the splenic marginal zone. Individual cytokines often have multiple effects, the results of which are dependent on their target cell. The variation of cytokines in our study coincided with the work of Shao et al. [25].

Other studies have confirmed that (expression/production?) of LPS-Induced TNF-alpha Factor (LITAF) was increased after APS inoculation, that it was secreted by B cells, and could activate macrophages and stimulate Th1 cells [22]. LITAF was chosen for inclusion in the current study because it was known to be derived from monocytes/macrophages and exerted potent cytotoxicity on tumorigenic cells [26]. It was previously shown that *Astragalus* could exert its anti-tumor effect on a variety of tumors *in vivo*, and that APS treatment could stimulate immune activities in rats with stomach cancer. The up-regulation of LITAF suggested that APS could prevent the formation of tumors in healthy bodies, while the down regulation of IFN-γ and LITAF suggested that the inflammatory activity was mediated.

In the present study, the expression levels of IL-2, IL-4, and IL-10 for APS treated groups was found to be markedly higher than for the control group after APS inoculation. Higher levels of IL-4 and IL-10 were produced pre- and post-challenge with H9N2 virus, suggesting that IL-4 and IL-10 induce long term immunity against H9N2 AIV infection (Figure 2 and 3). In this regard, APS most probably activated a network of interactions involving a number of different cytokines.

The humoral response is mediated by the secretion of antibodies by B lymphocytes, and antibody titer is a measure of the specific humoral immune response in chickens following vaccination [27]. In the current study, higher antibody titers were observed for the APS treated groups (particularly for the 5 and 10mg/kg groups) on d 7 and 14 following either H9N2 AIV infection or immunization with inactive vaccine. The results provided evidence that APS could enhance the humoral immune response in chickens, and that the 5 and 10mg/kg APS treatment groups were more effective in enhancing the immune response than the 20mg/kg APS treatment group. This observation corresponds with the work of Li et al. [27] who reported on the effectiveness of Chinese herbal medicines, including APS, in enhancing immunity in chickens. The antibody titer for the 5mg/kg APS group was the highest at 7 and 14 d post-immunization with the H9N2 inactivated vaccine. Collectively, the results obtained from this study indicated that the administration of the appropriate dose of APS could enhance antibody production and improve humoral immunity, and that APS had the properties of an anti-viral agent. These observations coincided with that reported by Kong et al. [28].

## Conclusions

From this study, the following conclusions were derived:

1. At an appropriate concentration (231.25μg/mL) APS can drastically reduce the proliferation of H9N2 virus.

2. APS enhanced the proliferation of CEF cells when used at concentrations > 9.766μg/mL. The exception, the simultaneous addition of APS and virus at APS concentrations of 2,500μg/mL and 1,250μg/mL.

3. APS effectively increases the expression of IL-2, IL-4, IL-6, IL-10, LITAF and IL-12, promotes cell growth, and enhances anti-H9N2 activity.

4. APS promoted a rapid humoral response following H9N2 vaccine immunization or H9N2 AIV infection.

5. The appropriate dose of APS (5 and 10mg/kg) significantly enhanced the specific immune response in chickens, and improved vaccine effectiveness; promoting an earlier peak that increased rapidly and was sustained for a longer period of time.

6. The CD_4_+, CD8+ T lymphocyte content and CD_4_^+^/CD_8_^+^ values for all the APS treatment groups were higher than those for the untreated (no APS) control group. The values for the 5 and 20mg/kg APS dose groups were significantly higher than the control group, which indicated that the appropriate dose of polysaccharide can promote the production of peripheral CD_4_+ and CD_8_+ lymphocytes in chickens, thereby enhancing cellular immunity.

7. APS inhibited H9N2 both *in vitro* and *in vivo*.

The precise mechanisms responsible for the response to APS require further examination. On the whole, APS has the potential to diminish disease progression in H9N2 infected chickens, and its use could provide alternative strategies for the control of H9N2 AIV infection.

## Competing interests

We declare that there is no competing interest from any source.

## Author contributions

Conceived and designed the experiments: ZYL, SK, QMX, XRL. Performed the experiments: SK, XRL, JJ JM.QYX, JYM, CYX. Analyzed the data: SK, XRL, JJ, QYX. Contributed reagents/materials/analysis tools: SK, XRL, JJ, JM. Manuscript authors: SK, XRL, ZYL, and QMX. All authors read and approved the final manuscript.
